# Self‐Reported Preparedness and Perceived Challenges in Managing Medical Emergencies Among Dentists at Komfo Anokye Teaching Hospital, Ghana: A Cross Sectional Study

**DOI:** 10.1002/puh2.70359

**Published:** 2026-09-04

**Authors:** Anabella Koteikai Kotey, Joseph Abu‐Sakyi, Manuella‐Krysty Asabea Siaw, John Billy Owusu Quarshie, Nana Atuahene Oti, Nana Bempong Owusu‐Ankomah, Samuel Baffour‐Awuah, Kwabena Opoku Owusu‐Ansah, Elijah Kwegyir Johnson, Kwame Adu Okyere Boadu

**Affiliations:** ^1^ School of Dentistry College of Health Sciences Kwame Nkrumah University of Science and Technology Kumasi Ghana; ^2^ Department of Health Policy Management and Economics School of Public Health College of Health Sciences Kwame Nkrumah University of Science and Technology Kumasi Ghana; ^3^ Komfo Anokye Teaching Hospital Kumasi Ghana; ^4^ Sunyani Teaching Hospital Sunyani Ghana; ^5^ University of Ghana Dental School, Korle Bu Accra Ghana

**Keywords:** dental clinic, emergency‐management training, medical emergencies, perceived challenges

## Abstract

**Background:**

Medical emergencies in dental settings are uncommon but potentially life‐threatening, and the response depends on practitioner training, institutional protocols and available equipment. Little has been published on these elements in Ghanaian dental services. This study described self‐reported emergency encounters, training status, protocol awareness, equipment availability and perceived challenges at one tertiary facility.

**Methodology:**

A descriptive cross‐sectional study was conducted at the Oral Health Directorate, Komfo Anokye Teaching Hospital, Kumasi. All 78 dentists on the roster were approached (attempted census); 62 returned completed self‐administered questionnaires over 14 consecutive working days (response rate 79.5%). All variables were categorical, summarised as frequencies and percentages. Associations of rank and years of practice with formal emergency‐management training status were examined using *χ* ^2^ tests with Yates’ correction and Fisher's exact test with odds ratios (ORs).

**Results:**

Percentages denote the proportion of 62 respondents reporting each multiple‐response item, not the frequency of events among patients. Hypoglycaemia (87.1%) and syncope (79.0%) were most often reported as ever encountered, then asthma attack (69.4%), seizures (62.9%) and anaphylaxis (48.4%). Thirty‐six (58.1%) reported formal training, 22 (35.5%) a written protocol and none an automated external defibrillator (AED). Training was reported more often by senior than junior dentists (69.2% vs. 39.1%; *p* = 0.040; OR 3.50, 95% confidence interval [CI] 1.19–10.29); the association with years of practice was borderline and test‐dependent (Yates‐corrected *p* = 0.052; Fisher's exact *p* = 0.039; OR 3.20, 95% CI 1.12–9.15). Commonest challenges were inadequate equipment (87.1%), lack of training (72.6%) and no clear protocol (69.4%).

**Conclusion:**

Two in five dentists reported no formal emergency‐management training, most were unaware of a written protocol, and none reported access to an AED. These self‐reported, single‐centre findings suggest that periodic training, written protocols and verified equipment stocks merit institutional attention; confirmation in larger multi‐centre studies is needed.

## Graphical abstract

Medical emergencies are uncommon in dental clinics but demand an immediate response. Among 62 dentists at a Ghanaian tertiary hospital, 41.9% reported no formal emergency training, 64.5% were unaware of a written protocol, and none reported a defibrillator in their clinic. Training was reported least often by the junior dentists.

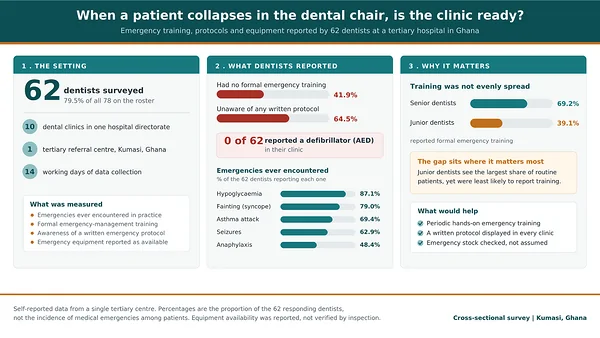

## 1 Introduction

Medical emergencies are fatal cases that ought to be identified and managed immediately when their symptoms present. In the dental setting, however, the current situation in resource‐constrained environments is not reflective of proactive emergency planning on the part of institutions [1]. Dentists in these settings normally rely on experiential improvisation without the benefit of clear protocols, which invariably leads to heightened patient risk when an emergency arises [1, 2]. The stakes are high: a single poorly managed emergency can be fatal for the patient and professionally devastating for the practitioner.

Routine dental procedures can be marred by unexpected medical emergencies. In worldwide statistics, the occurrence rate is approximately 0.1%–4.7% of all dental visits [3]. In one dental school clinic, 250 emergency cases were recorded over a 12‐year period, yielding a prevalence of 7.9% [4]. Another landmark study recorded 105 emergencies out of 3706 dental patients, giving a rate of 2.8% [5]. These figures, while seemingly small in isolation, are deceptive; even a 1% rate across the tens of thousands of dental visits recorded annually at a tertiary facility like Komfo Anokye Teaching Hospital (KATH) translates into a substantial absolute number of life‐threatening events.

The broader socioeconomic context of a healthcare system fundamentally shapes the resources available to manage such emergencies. Several structural equation modelling studies have established multidirectional relations between life course socioeconomic conditions and health outcomes, including oral health [6, 7]. These determinants of health apply equally to the dental emergency setting: poverty, institutional underfunding and inadequate human resource development translate directly into under‐equipped clinics and under‐trained practitioners [8, 9]. The interplay of these factors points clearly to the fact that preparedness is not merely about individual competence but the broader systemic environment in which dentists operate.

The readiness of dental practitioners to manage emergencies has been found to be worrying globally. More than half of surveyed practitioners in one international study cited lack of confidence as a key barrier to effective emergency response [10]. Earlier studies in developed countries pointed out that approximately 90% of dental practices in Great Britain were stocked with aspirators, airways, oxygen, adrenaline and injectable steroids, all crucial in the management of medical emergencies [11, 12]. The availability and accessibility of such resources in Ghanaian dental clinics, and in KATH specifically, were unknown prior to this study. Bridging this empirical gap is crucial for patient safety and institutional policy development.

KATH, situated in the Ashanti Region, is a 1200‐bed tertiary facility with ten dental clinics in its Oral Health Directorate. It is the second‐largest hospital in Ghana and serves as the primary referral centre for the middle belt of the country. The dental clinics at KATH receive a large volume of complex cases, and the risk of medical emergencies is correspondingly elevated. Despite this, there is a paucity of empirical evidence on emergency preparedness within these units. This study was therefore designed to assess the types of medical emergencies encountered, the level of preparedness and the challenges faced with a novel quantitative focus on whether preparedness is statistically associated with professional rank and years of clinical experience.

Practitioner readiness has been reported as suboptimal in several settings. More than half of the practitioners surveyed in one cross‐sectional study cited lack of confidence as a barrier to effective emergency response [10]. In Poland, 8.4% of dentists had never enrolled in any continuing professional development programme covering cardiopulmonary resuscitation [13], and in Nepal 46% had attended no post‐qualification emergency training [14]. Surveys from the United Kingdom conducted more than two decades ago indicated that approximately 90% of general dental practices stocked aspirators, airways, oxygen, adrenaline and injectable steroids [11, 12].

The Ghanaian evidence base does not permit an equivalent statement. Published local work has examined the patient side of dental care, including barriers to seeking treatment and health‐seeking behaviour for dental abscesses [15, 16], but no published Ghanaian study during our literature search had described what proportion of practising dentists had received formal emergency‐management training, whether written emergency protocols existed in their clinics, or which emergency items were actually stocked. This matters because the three determinants of an effective response, including training, protocol and equipment, are institutionally rather than individually determined, and none of them can be improved without first being measured. A further open question is whether training is evenly distributed across the workforce or concentrated among more senior practitioners, as an uneven distribution would place the least‐trained practitioners in the highest volume clinical roles.

This study was therefore designed to describe, among dentists at the Oral Health Directorate of KATH, the types of medical emergencies they reported having encountered, their formal emergency‐management training status, their awareness of a written emergency protocol, the emergency items they reported being available in their clinics and the challenges they perceived; and to examine whether formal emergency‐management training status was associated with professional rank and years of clinical practice.

## 2 Methods

### 2.1 Study Area

This study was conducted at the Oral Health Directorate of KATH, a 1200‐bed tertiary referral hospital in Kumasi that serves the Ghana and serves as the primary referral centre for the middle belt of Ghanathe country, serving a vast and diverse patient population. The directorate Oral Health Directorate, which is the site of this study, comprises ten operational dental clinics and represents the largest concentration of dental clinical activity in the Ashanti Region.

### 2.2 Study Design

A descriptive cross‐sectional design was used. A self‐administered questionnaire was employed to document, at a single point in time, dentists’ self‐reported previous encounters with medical emergencies during clinical practice, their formal emergency‐management training status, their awareness of a written departmental emergency protocol, the emergency equipment and medications they reported as available in their clinics, and the challenges they perceived in managing such emergencies. The design does not permit estimation of the incidence of medical emergencies among patients, nor inference about causality.

### 2.3 Study Population, Eligibility Criteria, Sampling and Sample Size

The study population comprised all dentists working at the Oral Health Directorate of KATH. Local human resource records listed 78 dentists at the time of the study. Because the accessible population was small and fully enumerable, no statistical sample size formula was applied; an attempted census was conducted, in which every eligible dentist on the roster was approached.

Eligibility criteria were as follows:

Inclusion criteria: (i) a licensed dentist employed at the Oral Health Directorate of KATH at the time of data collection, at any rank; and (ii) provision of written informed consent.

Exclusion criteria: (i) dentists on official leave (annual, study, sick or maternity leave) for the whole of the data collection window; and (ii) dentists who returned questionnaires that were incomplete for the demographic or outcome items.

Data were collected over 14 consecutive working days, from 12th August 2025 to 29th August 2025, with approximately five dentists approached per working day until the roster was exhausted. Of the 78 dentists listed, 62 consented and returned completed questionnaires, giving a response rate of 79.5%. The remaining 16 were either on official leave for the entire collection window or did not return a completed questionnaire.

### 2.4 Study Variables and Measurements

The variables measured were self‐reported and were of two kinds:

Descriptive variables: (i) Types of medical emergencies ever encountered during clinical practice at KATH, captured as a multiple‐response item listing seven conditions (syncope, hypoglycaemia, seizures, asthma attack, anaphylaxis, cardiac arrest and hypertensive crisis) with an open ‘other’ option; (ii) awareness of a written medical emergency protocol in the respondent's clinic (yes/no/not sure); (iii) availability of emergency equipment and medications, captured as a multiple‐response checklist of seven items and reported by the respondent rather than verified by the investigators; and (iv) perceived challenges in managing medical emergencies, captured as a multiple‐response item listing five challenges with an open ‘other’ option.

Outcome variable for inferential analysis: Formal emergency‐management training status was the sole outcome used in the inferential analysis and was defined as a binary variable: self‐reported receipt of formal training in the management of medical emergencies (yes/no). This single item captures one component of preparedness only. Because the questionnaire items on training, protocol awareness and equipment availability are conceptually distinct and the instrument was not designed or validated for scoring, no composite preparedness score was derived.

Explanatory variables: Sex, professional rank and years of clinical practice. Rank was collapsed into ‘senior’ (dental officers and specialists combined, *n* = 39) and ‘junior’ (house officers, *n* = 23). This grouping reflects the structure of the Ghanaian dental workforce, in which house officers are under supervision, whereas dental officers and specialists have completed housemanship and practise independently; the specialist category alone (*n* = 14) was too small to be analysed separately. Years of practice was collapsed into ‘more experienced’ (6–10 years and >10 years combined, *n* = 34) and ‘less experienced’ (<2 years and 2–5 years combined, *n* = 28). The 6‐year cut‐point was specified before analysis on three grounds: First, in Ghana the first post‐qualification year is spent in housemanship and the immediately following years in early independent practice, so that 6 years approximates the point by which a practitioner has completed housemanship, at least one full relicensure and continuing professional development cycle and, for those on the specialist pathway, substantial residency exposure; second, the four original categories produced a sparse cell (2–5 years, *n* = 5) that would have violated the expected‐count requirements of a 4 × 2 *χ* ^2^ test; and third, the cut‐point produced the most balanced dichotomy available (34 vs. 28), maximising statistical efficiency while preserving the ordinal structure of the original variable. The arbitrariness inherent in any dichotomisation is acknowledged in the limitations.

### 2.5 Data Collection Technique

Data were collected using a well‐structured, self‐administered printed questionnaire adapted from the validated instrument of Atherton et al. [11]. The following modifications were made for the Ghanaian dental context. First, the list of emergencies was reduced to the seven conditions most consistently reported in dental settings in Africa and other low‐ and middle‐income countries, and hypoglycaemia was retained as a listed option in view of the local diabetes burden and the common practice of prolonged fasting before clinical appointments. Second, items specific to United Kingdom general dental practice, including those relating to practice‐based sedation and general anaesthesia services, were removed, as these services are not delivered in the study clinics. Third, the original drug and equipment inventory was replaced with a seven‐item checklist restricted to items procured through the hospital's central stores and therefore realistically obtainable in the study setting. Fourth, a section on perceived challenges, which has no counterpart in the original instrument, was added and informed by local literature on service constraints. Fifth, response formats were simplified to binary or multiple‐response ticks, and terminology was localised to Ghanaian job titles (house officer, dental officer and specialist).

The questionnaire comprised four sections: Section A, background and demographic information; Section B, types of medical emergencies encountered; Section C, formal training status, protocol awareness and equipment availability; and Section D, perceived challenges. A pretest was conducted with five dentists at the dental clinic of the KNUST Hospital, a separate facility from the study site. Items identified as ambiguous were reworded to improve face validity, and pretest responses were not included in the analysis. All questionnaires were printed, distributed and collected by hand.

### 2.6 Quality Control

Completed questionnaires were checked for completeness at the point of collection. All data were double‐entered into Microsoft Excel, cleaned for inconsistencies and subsequently transferred to IBM SPSS Statistics version 27 for analysis. Discrepancies identified during data entry were resolved by reference to the original questionnaires.

### 2.7 Data Analysis

All variables analysed in this study were categorical. Sex, rank, years of practice, formal emergency‐management training status and protocol awareness were summarised as frequencies and percentages of the 62 respondents. The items on emergencies encountered, equipment availability and perceived challenges were multiple‐response items; for these, the frequency and the percentage of respondents endorsing each option are reported, with 62 as the denominator throughout, so that the percentages do not sum to 100. No continuous variables were analysed, and means and standard deviations were accordingly not computed for any variable.

For the inferential analysis, both the outcome (formal emergency‐management training status) and each explanatory variable (rank and years of practice) were binary following the categorisation described above, yielding 2 × 2 contingency tables. The *χ* ^2^ test of independence was therefore the appropriate test of association for these variable types. All expected cell counts exceeded five (minimum expected count 9.65 for rank and 11.74 for years of practice), satisfying the assumptions of the test. Yates’ continuity correction was applied because of the modest overall sample size, and Fisher's exact test was computed for both tables so that conclusions could be assessed for sensitivity to the choice of test. Odds ratios were calculated as the measure of effect size and are presented with 95% confidence intervals derived by the logit (Woolf) method; the phi coefficient is reported as a measure of the strength of association for these 2 × 2 tables. All tests were two‐sided with the significance threshold set at *α* = 0.05. No adjustment was made for multiple comparisons.

### 2.8 Ethical Considerations

Ethical clearance was granted by the Committee on Human Research, Publications and Ethics (CHRPE) of the KNUST School of Medicine and Dentistry (CHRPE AP/779/25). An introductory letter from the department was submitted to the Head of the Oral Health Directorate at KATH. Written informed consent was obtained from all participants prior to data collection. Participation was voluntary, and participants were informed of their right to withdraw at any time without consequence. All responses were anonymised, and no incentives were offered. The study was conducted in strict accordance with the principles of the Declaration of Helsinki.

## 3 Results

### 3.1 Demographic Characteristics of Respondents

Sixty‐two dentists participated. Most respondents were male (*n* = 39, 62.9%). By rank, dental officers formed the largest group (*n* = 25, 40.3%), followed by house officers (*n* = 23, 37.1%) and specialists (*n* = 14, 22.6%). The largest group by experience had 6–10 years of practice (*n* = 26, 41.9%), followed by those with less than 2 years (*n* = 23, 37.1%); 8 respondents (12.9%) had practised for more than 10 years (Table 1).

**TABLE 1:** Demographic characteristics of dentists at Komfo Anokye Teaching Hospital (KATH) (*n* = 62).

Variable/Category	Frequency (*n*)	Per cent
**Sex**		
Female	23	37.1
Male	39	62.9
**Rank**		
Dental officer	25	40.3
House officer	23	37.1
Specialist	14	22.6
**Years of practice**		
<2 years	23	37.1
2–5 years	5	8.1
6–10 years	26	41.9
>10 years	8	12.9

*Source:* Survey data, 2025.

### 3.2 Types of Medical Emergencies Reported as Encountered

Respondents were asked which medical emergencies they had ever encountered during clinical practice; the item permitted multiple responses, so the percentages represent the proportion of the 62 respondents reporting each condition and not the frequency of these events among patients attending the clinics. Hypoglycaemia was the most frequently reported condition, with 87.1% (*n* = 54) of respondents reporting at least one previous encounter, followed by syncope (79.0%, *n* = 49), asthma attack (69.4%, *n* = 43), seizures (62.9%, *n* = 39) and anaphylaxis (48.4%, *n* = 30). Hypertensive crisis (29.0%, *n* = 18) and cardiac arrest (27.4%, *n* = 17) were reported least often (Table 2).

**TABLE 2:** Types of medical emergencies reported as ever encountered by dentists at Komfo Anokye Teaching Hospital (KATH) (multiple‐response item, *n* = 62 respondents).

Medical emergency	Frequency (*n*)	Respondents reporting (%)
Hypoglycaemia	54	87.1
Syncope	49	79.0
Asthma attack	43	69.4
Seizures	39	62.9
Anaphylaxis	30	48.4
Hypertensive crisis	18	29.0
Cardiac arrest	17	27.4

*Source:* Survey data, 2025.

### 3.3 Formal Training Status, Protocol Awareness and Self‐Reported Equipment Availability

Thirty‐six respondents (58.1%) reported having received formal training in the management of medical emergencies, whereas 26 (41.9%) reported none. Regarding protocol awareness, 22 respondents (35.5%) reported that a written medical emergency protocol existed in their clinic, 18 (29.0%) reported that none existed, and 22 (35.5%) were not sure.

Equipment availability was reported by respondents and was not physically verified by the investigators; the figures below therefore describe perceived rather than audited availability. Blood pressure monitors (71.0%, *n* = 44) and first aid boxes (67.7%, *n* = 42) were the items most often reported as available, and glucometers were reported by half of respondents (50.0%, *n* = 31). Epinephrine (41.9%, *n* = 26), oxygen cylinders (33.9%, *n* = 21) and glucose tablets or gel (33.9%, *n* = 21) were each reported by fewer than half. No respondent reported the availability of an automated external defibrillator (AED) (Table 3).

**TABLE 3:** Formal emergency‐management training status, protocol awareness and self‐reported equipment availability among dentists at Komfo Anokye Teaching Hospital (KATH) (*n* = 62).

Variable/Category	Frequency (*n*)	Per cent
**Formal emergency‐management training received**		
Yes	36	58.1
No	26	41.9
**Awareness of a written emergency protocol**		
Yes	22	35.5
No	18	29.0
Not sure	22	35.5
**Equipment/medications reported as available**		
Blood pressure monitor	44	71.0
First aid box	42	67.7
Glucometer	31	50.0
Epinephrine	26	41.9
Oxygen cylinder	21	33.9
Glucose tablets/gel	21	33.9
Automated external defibrillator	0	0.0

*Source:* Survey data, 2025.

### 3.4 Association Between Demographic Characteristics and Formal Emergency‐Management Training Status

*χ* ^2^ tests of independence were performed on 2 × 2 contingency tables with formal emergency‐management training status as the outcome. Results are summarised in Table 4.

**TABLE 4:** Association of rank and years of practice with formal emergency‐management training status (*n* = 62).

Variable	Grouped category	df	*χ* ^2^ (Yates)	*p* value	Fisher's exact *p*	OR (95% CI)
Rank	Senior vs. junior	1	4.218	0.040	0.033	3.50 (1.19–10.29)
Years of practice	More vs. less experienced	1	3.777	0.052	0.039	3.20 (1.12–9.15)

*Note:* All expected cell counts exceeded five (minimum expected count 9.65 for rank and 11.74 for years of practice). Odds ratios compare the odds of reporting formal emergency‐management training in the first‐named group relative to the second.

Abbreviations: CI, confidence interval; OR, odds ratio.

*Source:* Survey data, 2025.

#### 3.4.1 Rank

Among senior dentists, 69.2% (*n* = 27) reported having received formal emergency‐management training, compared with 39.1% (*n* = 9) of house officers, a difference of 30.1 percentage points. There was evidence of an association between rank and formal training status (*χ* ^2^(1) = 4.218, *p* = 0.040; OR 3.50, 95% CI 1.19–10.29; phi = 0.29). Fisher's exact test gave a consistent result (*p* = 0.033). The confidence interval is wide, indicating that the magnitude of the difference is estimated imprecisely (Table 5).

**TABLE 5:** Rank by formal emergency‐management training status (*n* = 62).

Rank	Trained (*n*)	Not trained (*n*)	Total	% trained
Senior (dental officer + specialist)	27	12	39	69.2
Junior (house officer)	9	14	23	39.1
Total	36	26	62	58.1

*Note: χ* ^2^(1) = 4.218, *p* = 0.040 (Yates’ correction); Fisher's exact *p* = 0.033; OR 3.50, 95% CI 1.19–10.29.

*Source:* Survey data, 2025.

#### 3.4.2 Years of Practice

Among the more experienced group (≥6 years, *n* = 34), 70.6% (*n* = 24) reported formal training, compared with 42.9% (*n* = 12) of the less experienced group (*n* = 28); OR 3.20, 95% CI 1.12–9.15; phi = 0.28. The two tests did not agree: the Yates‐corrected *χ* ^2^ yielded *χ* ^2^(1) = 3.777, *p* = 0.052, marginally above the conventional threshold, whereas Fisher's exact test yielded *p* = 0.039. Because the conclusion depends on which test is applied, this finding is best described as a possible association of borderline statistical significance rather than a definitive one, and it requires confirmation in a larger sample (Table 6).

**TABLE 6:** Years of practice by formal emergency‐management training status (*n* = 62).

Years of practice	Trained (*n*)	Not trained (*n*)	Total	% trained
More experienced (≥6 years)	24	10	34	70.6
Less experienced (<6 years)	12	16	28	42.9
Total	36	26	62	58.1

*Note:* Yates‐corrected *χ* ^2^(1) = 3.777, *p* = 0.052; Fisher's exact *p* = 0.039; OR 3.20, 95% CI 1.12–9.15.

*Source:* Survey data, 2025.

### 3.5 Perceived Challenges in Managing Medical Emergencies

Challenges were captured as a multiple‐response item. Inadequate emergency equipment was reported by 87.1% (*n* = 54) of respondents, lack of training by 72.6% (*n* = 45) and the absence of a clear protocol by 69.4% (*n* = 43). Delayed response from the emergency team (40.3%, *n* = 25) and fear of medico‐legal consequences (33.9%, *n* = 21) were also reported (Table 7).

**TABLE 7:** Challenges reported by dentists in managing medical emergencies at Komfo Anokye Teaching Hospital (KATH) (multiple‐response item, *n* = 62 respondents).

Challenge	Frequency (*n*)	Respondents reporting (%)
Inadequate emergency equipment	54	87.1
Lack of training	45	72.6
No clear protocol	43	69.4
Delayed emergency team response	25	40.3
Fear of medico‐legal consequences	21	33.9

*Source:* Survey data, 2025.

## 4 Discussion

This study describes self‐reported emergency experience, formal training status, protocol awareness, reported equipment availability and perceived challenges among dentists at a single tertiary dental service in Ghana.

### 4.1 Ordering of Reported Emergencies

Hypoglycaemia was the condition most frequently reported as ever encountered (87.1%), ahead of syncope (79.0%). This ordering differs from most published series, in which syncope predominates [13, 17, 18, 19]. Two features of the present study plausibly account for the difference. The first is measurement: the studies cited above generally report events over a defined recent period or extract them from clinical records, whereas the present item asked whether a respondent had ever encountered each condition across an entire career. A career‐long recall window inflates all proportions and inflates most of those conditions that recur, which favours metabolic events over single dramatic ones. The 44.8% reported for hypoglycaemia in Saudi Arabia [20] and the approximately 16% reported in Poland [13] are therefore not directly comparable with the present figure. The second is case mix: KATH is a tertiary referral centre, and the rising prevalence of diabetes in Ghana, combined with the common practice of prolonged fasting before appointments, would be expected to increase the number of at‐risk patients presenting for treatment. D'Innocenzo et al. [4] similarly identified hypoglycaemia among the commoner events in a patient population aged 45–64 years. The high proportion reporting syncope (79.0%), against 46.3% in Poland [13] and 34.3% in Belgium [18], is open to the same recall‐window explanation, although the mechanism described by Malamed [21], in which procedural anxiety and pain trigger a vasovagal response, remains applicable. These proportions should not be read as incidence estimates.

### 4.2 Distribution of Formal Training Across the Workforce

Senior dentists were more likely than house officers to report formal emergency‐management training (69.2% vs. 39.1%; OR 3.50, 95% CI 1.19–10.29). The direction of this finding is consistent with the pattern reported elsewhere, in which post‐qualification emergency training accrues with seniority and continuing professional development participation: 8.4% of Polish dentists had never attended a CPR‐focused CPD programme [13], and 46% of Nepalese dentists had attended no post‐graduation emergency training [14]. The proportion untrained among KATH house officers (60.9%) is higher than both comparators, which suggests that the gap here is not simply the normal accumulation of training with time but a shortfall at the point of entry into clinical practice. Because house officers are the group with the greatest volume of unsupervised routine contact, this distribution places the least‐trained practitioners in the highest exposure roles.

The parallel association with years of practice (70.6% vs. 42.9%) points in the same direction but was borderline, with the two tests disagreeing (Yates *p* = 0.052; Fisher's exact *p* = 0.039) and a wide confidence interval (OR 3.20, 95% CI 1.12–9.15). It is therefore reported here as suggestive rather than established. Its interpretation is also less straightforward than that of rank, since training receipt is cumulative over a career and a positive association with experience is expected on arithmetic grounds alone. That expectation is a reason for caution: The more informative observation is that even among the more experienced group, 29.4% reported no formal training at all. Nogami et al. [22] documented rapid decay of basic life support skills among certified dentists in the absence of periodic refreshment, which indicates that ever having been trained is a weak proxy for current capability in either group. The present study measured training receipt only and did not assess competence, recency or content.

### 4.3 Convergence of Equipment, Training and Protocol Deficits

The three most frequently reported challenges—inadequate equipment (87.1%), lack of training (72.6%) and the absence of a clear protocol (69.4%)—mirror the objective findings from the same respondents, in which 41.9% reported no formal training and 64.5% either denied the existence of a written protocol or were unsure of it. This internal consistency between the perceived‐barrier items and the factual items strengthens both. Similar clustering of the three deficits has been documented in Nepal [14] and Brazil [23], which indicates that the pattern is not peculiar to this setting. The practical implication is that interventions directed at any single element are unlikely to be sufficient: Mehta et al. [14] found that although 43% of Nepalese dentists considered themselves able to manage emergencies, half of their clinics lacked emergency kits, so that individual capability could not be exercised.

The finding that no respondent reported an AED is the most consequential equipment result, given that 27.4% reported having encountered cardiac arrest. Vaughan et al. [19] reported limited AED availability internationally, and Pieren et al. [24] documented incomplete uptake even in well‐resourced settings, but a uniform nil return across a tertiary directorate is at the extreme of the published range. Rosenberg [25] treats the availability of emergency drugs and equipment as part of the standard of care rather than a discretionary provision. This finding must nonetheless be qualified: Availability was reported by respondents and not verified by inspection, so it is possible that a device exists somewhere in the directorate without the clinical staff being aware of it. Either interpretation, absence or unawareness has the same operational consequence during an arrest.

Delayed emergency team response (40.3%) and fear of medico‐legal consequences (33.9%) were reported less often but are consistent with the barriers described by Murray et al. [26] and with the 33% of Saudi practitioners who reported a fear of causing harm during cardiopulmonary resuscitation [27]. These are addressable through explicit institutional backing for good‐faith emergency intervention and clearer escalation pathways, though the present data can identify the concern only and cannot quantify its effect on response times.

## 5 Limitations

Some limitations qualify these findings. First, the study was conducted at a single tertiary institution, and the findings describe that directorate rather than dental practice in Ghana generally; district‐level and private clinics may differ substantially in both training access and equipment provision. Second, the cross‐sectional design captures a single point in time and cannot support causal inference about the observed associations. Third, all data were self‐reported. The item on emergencies encountered used a career‐long recall window and is therefore subject to recall bias, and reports of training and preparedness may be affected by social desirability bias. Equipment availability was reported by respondents and was not verified by physical inspection or stock audit, so reported availability may diverge from actual availability in either direction. Fourth, the design provides no patient‐level denominator. The percentages reported are proportions of responding dentists and cannot be interpreted as the prevalence or incidence of medical emergencies among patients attending the clinics. Fifth, the outcome analysed inferentially was a single binary item on receipt of formal training at any time. It carries no information on the recency, duration, content or quality of that training, nor on current competence, and no validated composite preparedness score was available.

## 6 Conclusion

In this single‐centre cross‐sectional survey, 41.9% of responding dentists reported having received no formal emergency‐management training, 64.5% either denied or were unsure of the existence of a written emergency protocol in their clinic, and no respondent reported access to an AED. Hypoglycaemia and syncope were the conditions most frequently reported as ever encountered, and inadequate equipment, lack of training and the absence of a clear protocol were the most frequently perceived challenges. Formal training was reported more often by senior dentists than by house officers, and a similar but borderline pattern was observed for years of practice. These findings are self‐reported, derived from one tertiary directorate, and describe training receipt rather than demonstrated competence; they should be regarded as indicating where institutional attention and further, better verified investigation are warranted, rather than as a definitive account of emergency preparedness in Ghanaian dental practice.

This study demonstrates the varying preparedness levels of dentists at KATH in managing medical emergencies encountered daily, and how these levels are significantly stratified by professional rank and accumulated clinical experience. Key findings are as follows: an alarming proportion of dentists, particularly house officers and early‐career practitioners, have not received adequate formal training; emergency protocols are either absent or inadequately disseminated; and the dental clinics lack critical emergency equipment, most notably AEDs. Rank was significantly associated with preparedness (*χ* ^2^(1) = 4.218, *p* = 0.040; OR = 3.50), and years of practice was equally significantly associated with preparedness (Fisher's exact *p* = 0.039; OR = 3.20). Both associations demonstrated moderate effect sizes and converged on the same conclusion that preparedness at KATH was not randomly distributed, but it was systematically tied to seniority. Inadequate equipment (87.1%), lack of training (72.6%) and the absence of clear protocols (69.4%) are the main barriers, compounded by delayed responses and medico‐legal fears. All these findings highlight the intricacies of institutional and clinical factors in driving emergency preparedness disparities at KATH, and they demand an urgent, coordinated institutional response.

## 7 Recommendations

Consistent with the qualifications noted above, the following are offered as suggestions arising from the findings.

On the basis of the findings of this study, the following recommendations are made:

- Periodic emergency‐management training, incorporating simulation‐based practical components in line with the evidence on skill retention [22, 28], could be considered for staff at the Oral Health Directorate, with particular attention to house officers and dentists in their first years of practice, who reported training least often.
- A review of emergency stocks across the ten dental clinics, verified by physical inspection rather than by report, would establish actual availability of oxygen, epinephrine, glucometers, glucose preparations and first aid supplies and would clarify whether an AED is accessible to the directorate.
- A written, standardised emergency protocol covering the conditions most frequently reported, displayed in each clinic and reinforced through periodic drills, would address the finding that most respondents were unaware of any such protocol.
- Periodic institutional audit of training coverage, equipment functionality and protocol awareness would allow these indicators to be monitored over time.
- Further research is warranted, including multi‐centre studies covering district and private dental facilities, studies that verify equipment directly and assess competence rather than training receipt, and prospective record‐based work capable of estimating the actual incidence of medical emergencies in Ghanaian dental settings.

## Author Contributions

**Anabella Koteikai Kotey**: conceptualization, methodology, resources, visualization. **Joseph Abu‐Sakyi**: supervision, methodology, resources, validation. **Manuella‐Krysty Asabea Siaw**: data collection, visualization. **John Billy Owusu Quarshie**: formal analysis, writing – original draft preparation. **Nana Atuahene Oti**: formal analysis, writing – original draft preparation. **Nana Bempong Owusu‐Ankomah**: formal analysis, writing – original draft preparation. **Samuel Baffour‐Awuah**: formal analysis, writing – original draft preparation. **Owusu‐Ansah Kwabena Opoku**: validation, formal analysis, writing – original draft preparation. **Elijah Kwegyir Johnson**: writing – original draft preparation, writing – review and editing. **Kwame Adu Okyere Boadu**: methodology, writing – original draft preparation, writing – review and editing.

## Funding

The authors have nothing to report.

## Disclosure

All authors have read and approved the final version of the manuscript. Kwame Adu Okyere Boadu had full access to all the data in this study and takes complete responsibility for the integrity of the data and the accuracy of the data analysis.

## Conflicts of Interest

The authors declare no conflicts of interest.

## Transparency Statement

Kwame Adu Okyere Boadu affirms that this manuscript is an honest, accurate and transparent account of the study being reported; that no important aspects of the study have been omitted; and that any discrepancies from the study as planned have been explained.

## Data Availability

The data that support the findings of this study are available from the corresponding author upon reasonable request.
